# Advancing multi-database cohort studies: lessons learned from a studyathon in a pharmaceutical company

**DOI:** 10.1093/jamiaopen/ooag189

**Published:** 2026-09-30

**Authors:** Elif Inan-Eroglu, Daloha Rodriguez-Molina, Khaled Abdelgawwad, Carina Dinkel-Keuthage, Montse Soriano Gabarró, Pareen Vora, Gianmario Candore, David Vizcaya, Katrin Manlik

**Affiliations:** Pharmaceuticals, Global Medical & Evidence, Bayer AG, 13353 Berlin, Germany; Pharmaceuticals, Global Medical & Evidence, Bayer AG, 13353 Berlin, Germany; Pharmaceuticals, Global Medical & Evidence, Bayer AG, 13353 Berlin, Germany; Pharmaceuticals, Global Medical & Evidence, Bayer AG, 13353 Berlin, Germany; Pharmaceuticals, Global Medical & Evidence, Bayer AG, 13353 Berlin, Germany; Current affiliation: Independent Researcher, 14482 Potsdam, Germany; Pharmaceuticals, Global Medical & Evidence, Bayer AG, 13353 Berlin, Germany; Pharmaceuticals, Global Medical & Evidence, Bayer AG, 13353 Berlin, Germany; Pharmaceuticals, Global Medical & Evidence, Bayer Hispania SL, 08970 Sant Joan Despí, Spain; Current affiliation: Alexion Pharmaceuticals SL, 08029 Barcelona, Spain; Pharmaceuticals, Global Medical & Evidence, Bayer AG, 13353 Berlin, Germany

**Keywords:** studyathon, real-world data, real-world evidence, OHDSI, OMOP common data model

## Abstract

**Objectives:**

To evaluate the feasibility and applicability of the studyathon format within a pharmaceutical company setting for generating reliable real-world evidence rapidly for a business-relevant research question.

**Materials and methods:**

Conducted at Bayer, the studyathon assessed recurrent events following a first-ever transient ischemic attack. It involved an 8-week preparatory phase, a 1-week execution phase, and a 3-week post-event phase, using multiple real-world data sources.

**Results:**

The studyathon concluded successfully with a conference abstract submission. Key learnings included the importance of defining precise research questions, organizational alignment, thorough preparation, and leveraging diverse expertise and skill sets.

**Discussion:**

The studyathon format proved suited for a pharmaceutical setting and revealed insights into its application. Our learnings highlight differences from multi-stakeholder community studyathons.

**Conclusion:**

The studyathon demonstrated its potential as a valuable research approach within a pharmaceutical company. It improved study timelines and cross-functional collaboration while preserving scientific rigor.

## Background

The Observational Medical Outcomes Partnership (OMOP) Common Data Model (CDM) was developed as a standardized framework to ease challenges of generating reliable evidence in a timely manner from multiple real-world data (RWD) sources. It transforms diverse healthcare databases into a common format, standardizes the terminology used, and therefore enables researchers to conduct large-scale observational studies with consistency and reproducibility across diverse data sources.^1^ The model is maintained by the Observational Health Data Sciences and Informatics (OHDSI) initiative, a global, multi-stakeholder, interdisciplinary collaborative which aims to improve health outcomes by empowering a community to collaboratively generate evidence from RWD. Through shared standardized analytical tools, methodologies, and data standards, OHDSI fosters transparent research practices that increase the reliability of observational studies.^1^^,^^2^

A studyathon is an innovative research approach akin to a hackathon, applied specifically to address clinical research questions. Studyathons developed by OHDSI focus on conducting observational studies to generate evidence within a short timeframe. This approach involves a focused, multiday research event where a multidisciplinary team collaborates intensively to generate reliable scientific evidence using harmonized RWD, analytical tools, and methods.^2^ Studyathons can support various phases of observational research, including the ideation phase for hypothesis generation, the design phase for protocol development and feasibility, the execution phase including data analysis, and the dissemination phase for results interpretation and publication preparation.^2^

Several studyathons have been conducted within the OHDSI community and in collaboration with the European Health Data and Evidence Network (EHDEN), which has built a federated network of healthcare data sources standardized to the OMOP-CDM in Europe.^1^^,^^3^ These community-driven multi-stakeholder studyathons typically involved multiple research institutions, pharmaceutical companies, data partners, as well as academic collaborators.^4–9^ However, to date, no studyathon has been documented within a single pharmaceutical company setting which highlights a gap in applying this study approach to pharma-company-specific research.

## Objectives

In July 2024, we conducted the first studyathon at Bayer to assess Recurrent Events and Characteristics following a first-ever transient ischemic attack (REACT-TIA) through a retrospective multi-database cohort study.^10^ Our objective was to test the feasibility and applicability of the studyathon format within a single-entity pharmaceutical company setting while rapidly generating reliable evidence for a business-relevant research question. Unlike multi-stakeholder community studyathons, Bayer’s studyathon was conducted entirely within 1 organization. Proposed potential research questions were prioritized according to predefined criteria, including scientific relevance, feasibility, data availability, and strategic business alignment. Based on this evaluation, 1 research question was selected, which aimed to describe the characteristics and incidence of ischemic stroke in patients who had experienced a first non-cardioembolic transient ischemic attack. Since thrombosis and stroke are well-known therapeutic and scientific areas at Bayer,^11–13^ this topic is highly relevant to the company’s goals. Additionally, the availability of OMOP-compatible tools and multiple in-licensed data sources in OMOP format supported the feasibility of conducting this study within the short timeframe of a studyathon. In this report, we share our learnings to help various stakeholders, including organizations from industry, academia, healthcare, policy, and public health, to replicate and optimize the studyathon format for rapid and reliable large-scale evidence generation.

## Materials and methods

The studyathon included an 8-week preparatory phase, a 1-week execution phase, and a 3-week post-event phase. It involved the use of multiple in-licensed RWD sources in OMOP format, as well as OHDSI and Data Analysis and Real World Interrogation Network (DARWIN EU) tools.

### Preparatory phase

This phase built a robust foundation for the studyathon. It involved a core team of 7 colleagues with prior expertise in conducting studies with OMOP methodologies and OHDSI standard analytical tools, specialized skills in the therapeutic area, research question formulation, project management, as well as epidemiological and medical knowledge.

First, we focused on defining the research question and strategically coordinated across internal functions to achieve alignment of the study objectives. This approach also considered the feasibility of addressing these objectives based on the selected data sources (MarketScan^®^ and Optum’s de-identified Clinformatics^®^ Data Mart Database) and aimed to engage additional participants with diverse skill sets.

We created 4 sub-teams with specific responsibilities^2^: (1) coordination and communication, tasked with managing logistics, and internal communications to promote collaboration; (2) phenotyping, responsible for defining and validating clinical phenotypes and cohorts; (3) medical writing, focused on drafting the study protocol, reports, and documentation; and (4) feasibility and execution, responsible for assessing data availability, analytical feasibility, and planning the execution workflow (Figure 1).

**Figure 1.**
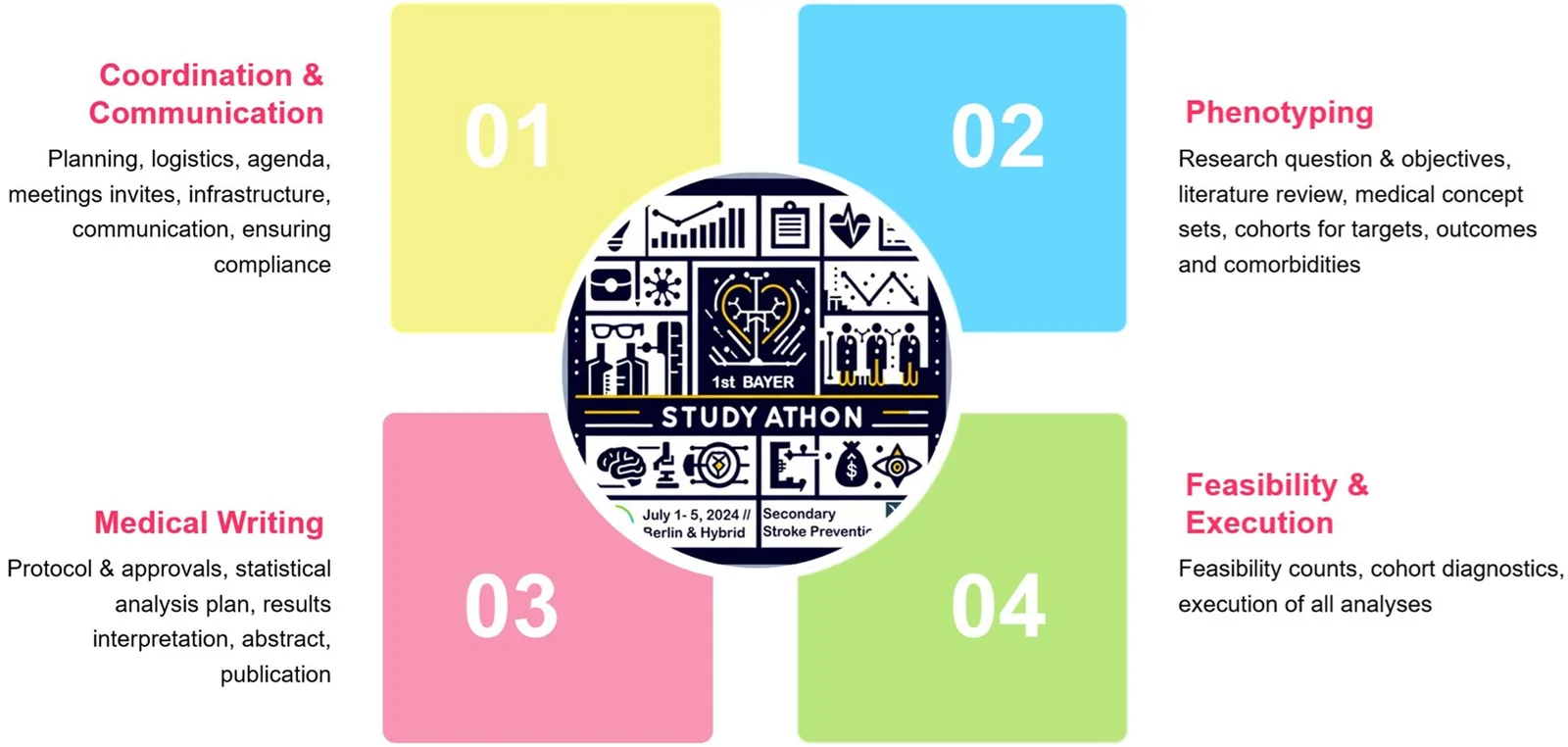
Organizational setup of the studyathon team.

This structure facilitated the preparation of all necessary steps. Additional feasibility work included securing data access, setting up analytical environments, and conducting training sessions on OHDSI tools. A comprehensive preparatory checklist was created to ensure no critical steps were overlooked (Supplementary File 1).

### Execution phase

The execution phase was conducted over 1 week in a hybrid setting, allowing 18 participants to join onsite or remotely from different countries and time zones. Each participant was expected to commit between 2 and 8 hours daily. Key activities included data extraction and cohort characterization, statistical analysis, iterative refinement of phenotypes based on preliminary results, and drafting initial findings for discussion. Analytical tools such as ATLAS^14^ for cohort definition and exploration, and analytical packages from OHDSI^15^ (eg, CohortDiagnostics) and DARWIN EU^16^ (eg, IncidencePrevalence) were used for standardized and reproducible analyses.

We prepared a detailed agenda to outline the daily goals and activities (Figure 2). A deliverables dashboard was implemented to track the progress of all interim and final study deliverables (ie, protocol, approvals, medical codelists/phenotypes, cohorts, analysis results, visualizations, interpretations, and documentation), which provided transparency and accountability and ensured that key milestones were met (Supplementary File 2). Throughout the studyathon week, we captured lessons learned at the end of each day to suggest improvements and provide insights for future studyathons. At the end of the week, we held “Big Medical Round Table” sessions, which brought together clinical, pharmacovigilance, and epidemiological experts to discuss and interpret the findings and evaluate the results from various perspectives.

**Figure 2.**
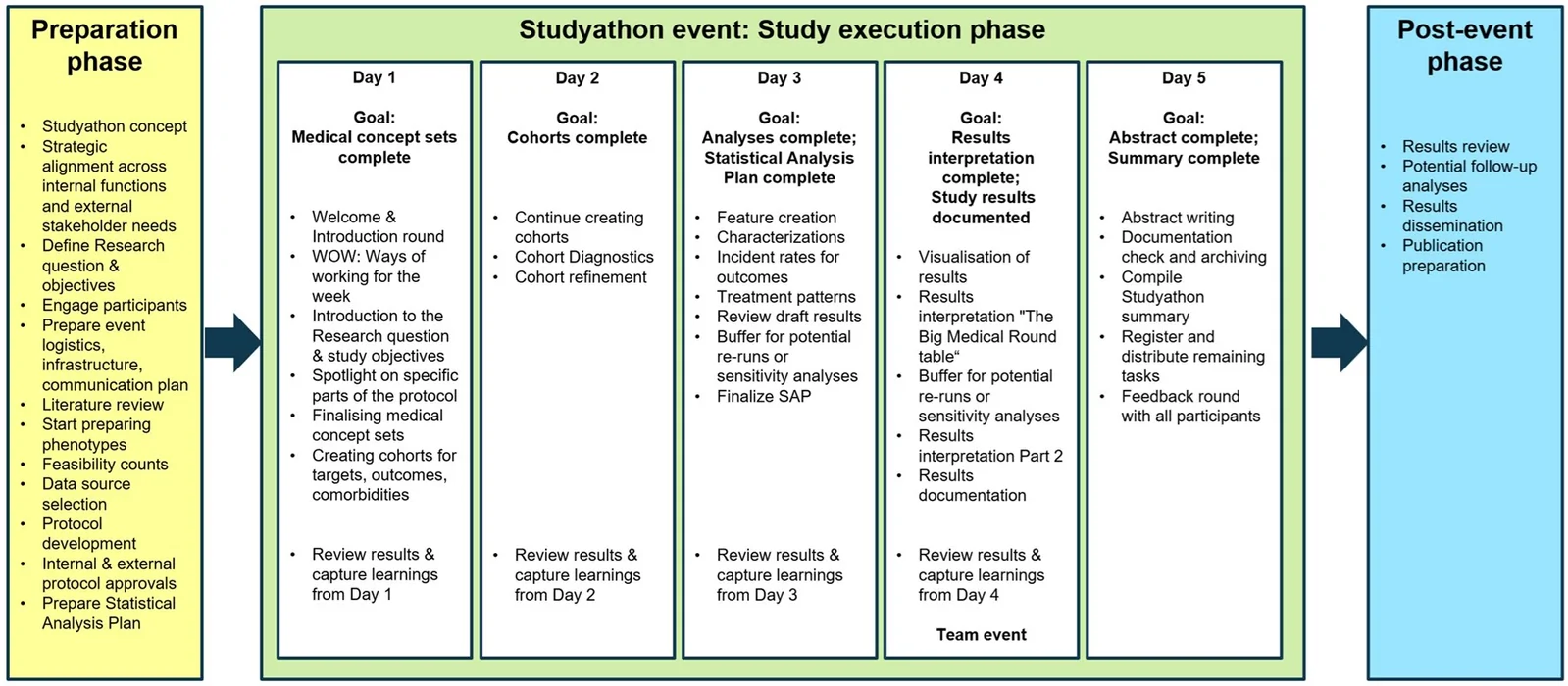
Agenda for the studyathon. Abbreviations: SAP, statistical analysis plan; WOW, ways of working.

### Post-event phase

The 3-week post-event phase focused on finalizing deliverables and disseminating outcomes. Responsibilities for post-event activities were assigned at the end of the execution week to maintain continuity. Activities included finalizing data analyses, preparing comprehensive reports, addressing any unresolved interpretative questions, and finalizing abstracts for external sharing. A checklist for follow-up work was created to guide these efforts (Supplementary File 1).

## Results

The studyathon was completed within a total timeline of 12 weeks. During the 1-week execution phase, the 18 participants established or reviewed 40 medical codelists, configured and tested 5 target and 5 outcome cohorts, executed 20 analyses in each of the 2 included data sources, developed 1 results dashboard, and authored 3 study documents (Supplementary File 2). The event concluded successfully with a compiled conference abstract that was submitted for the International Stroke Conference 2025 and the OHDSI Europe Symposium 2025.^8^^,^^17^ The studyathon provided several key learnings that were important for the success of the REACT-TIA study and will support the successful implementation of future events:

1. *Defining the research question*: clearly defining a well-scoped and precise research question is crucial. The intensity of the work required a strong focus on key deliverables. We suggest focusing on a single primary study objective while keeping secondary objectives to a minimum and refraining from any exploratory objectives to maintain clarity and efficiency.
2. *Organizational alignment*: ensure clear value proposition and strategic alignment of all internal stakeholders on project objectives when initiating a studyathon as with any study.
3. *Understanding database characteristics and context in advance*: thorough familiarity with data sources and existing literature was essential. This preparation enabled the team to rapidly interpret results and complete the abstract in time for the deadline without the delays typical of standard research cycles.
4. *Standardized analytics and execution strategy*: challenges in writing and executing analytical code can affect the feasibility of a studyathon. The use of standardized analytical tools supported efficient, reproducible analyses, while choosing a computationally manageable research question was key to completing the work within the limited timeframe.
5. *Sub-team structure and coordination*: having 4 sub-teams resulted in a fair distribution of workload, established clear deliverables for each group, and increased overall engagement. We recognized the necessity of appointing a single coordinator to oversee all logistics and activities, as well as a designated reference point for addressing all scientific questions.
6. *Adequate time for approvals*: planning sufficient time for protocol approvals was key, since the studyathon’s execution week was fixed in advance. Unlike standard research where administrative delays are often negligible, the immovable schedule means that any slippage in governance approval is unacceptable.
7. *Diverse expertise as a success factor*: bringing together diverse expertise was one of the key success factors and resulted in substantive cross-functional discussions that integrated clinical, epidemiological, pharmacovigilance, and data science perspectives and promoted shared learning among participants. Studyathon organizers must ensure the right skill sets are present.
8. *Daily lessons-learned sessions*: a daily lessons-learned session during the execution week provided participants with the chance to reflect on their experiences and suggest improvements for immediate implementation.
9. *Choice between onsite and hybrid setup*: most attendees participated in person, and the hybrid setup effectively engaged additional participants. However, it posed challenges in maintaining consistent engagement and effective communication across time zones and locations. Whenever feasible, in-person participation is recommended, as it fosters faster decision-making, more dynamic discussions, and smoother coordination among team members.
10. *Learning opportunities*: all participants gained plenty of hands-on experience with OHDSI tools and OMOP-CDM, which significantly increased their technical and methodological skills in RWD analysis. Participants also reported increased understanding of the cross-functional collaboration within the pharmaceutical company setting, as well as improved knowledge of thrombosis and stroke research. These learning opportunities not only contributed to personal and professional growth but also built internal capability for future studyathons.

## Discussion

The successful execution of the first studyathon at Bayer provided valuable insights into the adaptation of this innovative research format within a pharmaceutical company setting. It also demonstrated its potential applicability to other single-institution environments such as hospitals, regional research organizations, or academic groups.

Key distinctive features between community and internal pharma company studyathons lie in their scope, objectives, and operational constraints. Multi-stakeholder community studyathons, often facilitated by collaborative networks like OHDSI or EHDEN, involve multiple institutions and data partners, focus on research questions of general interest (eg, COVID-19) and have an emphasis on methodological advancement.^5^^,^^8^ In contrast, single-entity studyathons, such as the one conducted by Bayer, are organized within a single organization and are typically aligned with specific business or therapeutic priorities. Furthermore, company settings may impose stricter confidentiality and regulatory requirements, necessitating additional approvals compared to the more open frameworks of academic initiatives.

An essential prerequisite for successfully conducting this studyathon was the availability of a mature OHDSI and OMOP ecosystem. This included a robust IT infrastructure, an operational OHDSI instance with standard analytical packages, and WebAPI connectivity to OMOP databases. From a data perspective, continuous availability of analysis-ready OMOP data is required. This entails transformation into the OMOP-CDM through maintained extract, transform, and load pipelines, updates following database refreshes, routine data quality checks, and regular medical vocabulary updates.

The studyathon is particularly well-suited for certain types of studies and research questions.^2^ Multi-database retrospective cohort studies such as the REACT-TIA study are well-suited for contexts requiring standardized data models. These studies make use of existing RWD, which can be analyzed efficiently within a constrained timeframe, making them advantageous for time-limited research efforts. Descriptive analyses requiring rapid evidence generation, such as characterization of patients, post-marketing surveillance or exploratory analyses for hypothesis generation, are also appropriate.^2^^,^^18^ However, the suitability of a study for a studyathon is determined by its reliance on standardized analytical tools and methodologies (eg, OHDSI or DARWIN tools, OMOP-CDM) rather than the complexity of the research question, as the intense, time-limited format is not conducive to bespoke programming.

The focused, collaborative studyathon setting, in which all participants were dedicated to the study and readily available for review, discussion, and alignment, enabled completion of the execution phase within 1 week. Under routine operational conditions, the same volume of specification, analysis execution, documentation, and cross-functional alignment work would likely have required several weeks, given fewer resources and competing commitments and responsibilities.

The intensity of work during the execution week requires participants to dedicate significant time to achieve deliverables within a short time window. This can be both a motivator and a challenge.^2^^,^^18^ To maintain engagement, we found that a structured daily agenda with clear goals, regular progress updates via a dashboard, and daily lessons-learned sessions were effective in keeping participants focused and motivated. Fostering a collaborative environment by leveraging diverse expertise such as combining clinical, epidemiological, and data science perspectives encouraged active participation and improved discussions.

The choice between onsite and hybrid setups warrants careful consideration. In our studyathon, most participants attended in person, which facilitated direct interaction, rapid problem-solving, and a sense of camaraderie. The hybrid setup allowed inclusion of additional remote participants across different time zones, broadening access to expertise.

## Conclusion

Organizing the studyathon in 3 separate phases provided clear structure, speed, and focus. The approach followed was well-suited to the studyathon format, facilitating a rapid study execution while maintaining scientific rigor. Implementation of studyathons is feasible and proved valuable for sporadically addressing urgent evidence needs within a pharmaceutical company, adherence to tight study timelines and enhancing cross-functional collaboration.

## Supplementary Material

ooag189_Supplementary_Data

## Data Availability

No new datasets were generated or analyzed for this article. The underlying REACT-TIA study used in-licensed real-world data sources, which are not publicly available due to licensing restrictions.
