# Supplementary material for: Advancing multi-database cohort studies: lessons learned from a studyathon in a pharmaceutical company
Source: JAMIA Open. 2026 Sep 30;9(5):ooag189. doi: 10.1093/jamiaopen/ooag189 (PMC13625018; doi:10.1093/jamiaopen/ooag189)
Supplement: Supplementary file 1 [file ooag189_supplementary_data.zip › Supplementary File 1.pdf]

Supplementary Material 1: Checklist for preparatory work

The following preparatory activities shall be completed before the studyathon during the preparation phase (approx. 2 months)

- Setup organizational team
- Develop a time schedule (Preparation, Execution, Post-event phase)
- Define research question
  - Align with all stakeholders
- Conduct feasibility and select fit-for-purpose data sources
- Confirm studyathon event date
  - Consider locations of potential participants, including local holidays
  - Decide options for participation (onsite, hybrid, virtual)
- Promote the studyathon event and engage participants
  - Distribute announcement and event invite early before
  - Survey interested participants on background, skills and expectations
  - Conduct a studyathon info session
  - Design a logo for the studyathon event
  - Assign roles as needed and make sure all roles are sufficiently staffed with the necessary skills and resources
- Conduct literature review
- Develop and approve protocol
  - Develop draft
  - Obtain approvals within the organization
  - Obtain approvals outside the organization, if applicable, by data owners and/or data partners
- Prepare infrastructure
  - Ensure access for participants to relevant data sources

    - Room reservations (big video screen if hybrid event, consider options for breakout sessions and sub-team work, test infrastructure in room)
    - Test IT infrastructure (e.g., performance tests, access to analytical tools, availability of relevant standard analytical packages, access to databases)
    - setup separate sessions for each of the agenda items to allow break-out groups and participants attending particular sessions
    - Setup a Teams channel as main communication channel
- Prepare agenda
    - Consider locations and time zones of participants
- Prepare a deliverables dashboard for tracking task progress
- Selection of potential target conferences or journals for publication of study results before the studyathon event. Consider target audience, stakeholder needs, submission timelines, number of authors.

Supplementary Material 2: Checklist for follow-up work

The following closing activities shall be completed after the studyathon during the follow-up phase (approx. 4-6 weeks)

- ☐ Finalize documentation of results
- ☐ Communicate results to stakeholders
- ☐ Conduct lessons learned session
- ☐ Approvals and submission of abstract or manuscript
- ☐ Create Study Report
- ☐ Archiving
