# Supplementary material for: Advancing multi-database cohort studies: lessons learned from a studyathon in a pharmaceutical company
Source: JAMIA Open. 2026 Sep 30;9(5):ooag189. doi: 10.1093/jamiaopen/ooag189 (PMC13625018; doi:10.1093/jamiaopen/ooag189)
Supplement: Supplementary file 1 [file ooag189_supplementary_data.zip › Supplementary File 2 - REACT-TIA study.pdf]

REACT-TIA study: Studyathon Deliverables Dashboard

Medical Codelists (Concept Sets)	Status
N=40	
MC1 - AF	Delivered
MC2 - TIA	Delivered
MC3 - Ischemic Stroke	Delivered
MC4 - Intracranial bleeding	Delivered
MC5 - Hospitalization-related Bleeding	Delivered
MC6 - Hypertension	Delivered
MC7 - Myocardial Infarction (MI)	Delivered
MC8 - Anemia	Delivered
MC9 - Stroke	Delivered
MC10 - Hemorrhagic stroke	Delivered
MC11 - Hyperlipidemia	Delivered
MC12 - Chronic kidney disease (CKD)	Delivered
MC13 - Heart Failure	Delivered
MC14 - Chronic Coronary Artery Disease (CAD)	Delivered
MC15 - Peripheral Artery Disease (PAD)	Delivered
MC16 - Diabetes mellitus type-1 and type-2	Delivered
MC17 - Cancer excluding non-melanoma skin cancer	Delivered
MC18 - Dementia	Delivered
MC19 - Venous thromboembolism (VTE)	Delivered
MC20 - Mechanical heart valves	Delivered
MC21 - Congenital heart disease	Delivered
MC22 - Patent foramen ovale	Delivered
MC23 - History of Ischemic Stroke	Delivered
MC24 - History of hemorrhagic stroke	Delivered
M1 - Antihypertensives	Delivered
M2 - Antidiabetics	Delivered
M3 - Statins	Delivered
M4 - NSAIDs	Delivered
M5 - Antiplatelet and antithrombotic medications	Delivered
M6 - Aspirin	Delivered
M7 - Clopidogrel	Delivered
M8 - Ticagrelor	Delivered
M9 - Prasugrel	Delivered
M10 - Cilostazol	Delivered
M11 - Other Antiplatelets	Delivered
Oral anticoagulants	
M 12 - Warfarin	Delivered
M13 - Direct oral anticoagulants	Delivered
M14 - Rivaroxaban	Delivered
M15 - Dabigatran	Delivered
M16 - Apixaban	Delivered

Step 1: Create MC sets that are needed for the main cohorts
Step 2: Setup main cohorts TC1 and TC 2
Step:3 Further MC sets that are needed for Characterization

Feature Analyses	Status
N=50	
FA1 - AF	Delivered
FA2 - TIA	Delivered
FA3 - Ischemic Stroke	Delivered
FA4 - Intracranial bleeding	Delivered
FA5 - Hospitalization-related Bleeding	Delivered
FA6 - Hypertension	Delivered
FA7 - Myocardial Infarction (MI)	Delivered
FA8 - Anemia	Delivered
FA9 - Stroke	Delivered
FA10 - Hemorrhagic stroke	Delivered
FA11 - Hyperlipidemia	Delivered
FA12 - Chronic kidney disease (CKD)	Delivered
FA13 - Heart Failure	Delivered
FA14 - Chronic Coronary Artery Disease (CAD)	Delivered
FA15 - Peripheral Artery Disease (PAD)	Delivered
FA16 - Diabetes mellitus: type-1 and type-2 diabetes mellitus	Delivered
FA17 - Cancer, excluding non-melanoma skin cancer	Delivered
FA18 - Dementia	Delivered
FA19 - Venous thromboembolism (VTE)	Delivered
FA20 - Mechanical heart valves	Delivered
FA21 - Congenital heart disease	Delivered
FA22 - Patent foramen ovale	Delivered
FA23 - History of Ischemic Stroke	Delivered
FA24 - History of hemorrhagic stroke	Delivered
FA25 -Antihypertensives	Delivered
FA26 -Antidiabetics	Delivered
FA27 -Statins	Delivered
FA28 - NSAIDs	Delivered
FA29 - Antiplatelet and antithrombotic medications	Delivered
FA30 - Aspirin	Delivered
FA31 - Clopidogrel	Delivered
FA32 - Ticagrelor	Delivered
FA33 - Prasugrel	Delivered
FA34 - Cilostazol	Delivered
FA35 - Other Antiplatelets	Delivered
Oral anticoagulants	
FA36 - Warfarin	Delivered
FA37 - Direct oral anticoagulants	Delivered
FA38 - Rivaroxaban	Delivered
FA39 - Dabigatran	Delivered
FA40 - Apixaban	Delivered
FA41 - Age groups (18-30; 31-40; 41-50; 51-60; 61-70; 71-80; 81-9	Delivered
FA41 - Age groups (below and above 65)	Delivered
FA42 - Index year (pre and post COVID 19)	Delivered
FA43 - Estimated Glomerular Filtration Rate	Delivered
FA44 - Estimated Glomerular Filtration Rate - groups	Delivered
FA45 - Loop diuretics	Delivered
FA46 - Thiazides and other non-loop diuretics	Delivered
FA47 - Beta-blockers	Delivered
FA48 - Calcium-channel blockers	Delivered
FA49 - RAASi	Delivered
FA50 - History of stroke	Delivered

Cohorts - Target and Outcomes	Status
N=10	
TC1 - TIA - without evidence of AF or any prior TIA event	Delivered
TC2 - TIA_SAPT - SAPT between days 0 and 15 after the TIA	Delivered
TC3 - TIA_DAPT - DAPT in the 0 to 15 days after the TIA	Delivered
TC4 - TIA_NOAPT - no SAPT or DAPT in the 0 to 15 days after th	Delivered
TC5 - TIA_ANYSAPTORDAPT - either SAPT or DAPT in the 0 to 1	Delivered
OC1 - Ischemic stroke	Delivered
OC2 - Intracranial bleeding	Delivered
OC3 - Hospitalization-related bleeding	Delivered
OC4 - New-onset atrial fibrillation	Delivered
OC5 - Recurrent TIA	Delivered

DAPT definition: 2 Antiplatelets, sort order is not looked at

Analyses / Dashboard	Status
N=20	
A1 - TC1 Characterisation	Delivered
A2 - TC2 Characterisation	Delivered
A3 - TC3 Characterisation	Delivered
A4 - TC4 Characterisation	Delivered
A5 - TC5 Characterisation	Delivered
IR1 - Incidence rate analysis OC1 on	Delivered
IR2 - Incidence rate analysis OC2 on	Delivered
IR3 - Incidence rate analysis OC3 on	Delivered
IR4 - Incidence rate analysis OC4 on	Delivered
IR5 - Incidence rate analysis OC5 on	Delivered
TR1 - Treatment pathways TC1	Delivered
TR2 - Treatment pathways TC2	Delivered
TR3 - Treatment pathways TC3	Delivered
TR4 - Treatment pathways TC4	Delivered
TR5 - Treatment pathways TC5	Delivered
CD1 - TC1 CohortDiagnostics	Delivered
CD2 - TC2 CohortDiagnostics	Delivered
CD3 - TC3 CohortDiagnostics	Delivered
CD4 - TC4 CohortDiagnostics	Delivered
S1 - ShinyApp/Results dashboard	Delivered

Documents	Status
N=8	
D1 - Study classification	Delivered
D2 - Protocol	Delivered
D3 - PASS assessment	Delivered
D4 - Clinical Trial Posting assessment	Delivered
D5 - eRAP for CPRD protocol approval	Delivered
D6 - Statistical Analysis Plan (SAP)	Delivered
D7 - Abstract	Delivered
D8 - OS Study report	Delivered

Supplementary Information on the study design of the REACT-TIA study

1. Study Design Diagram

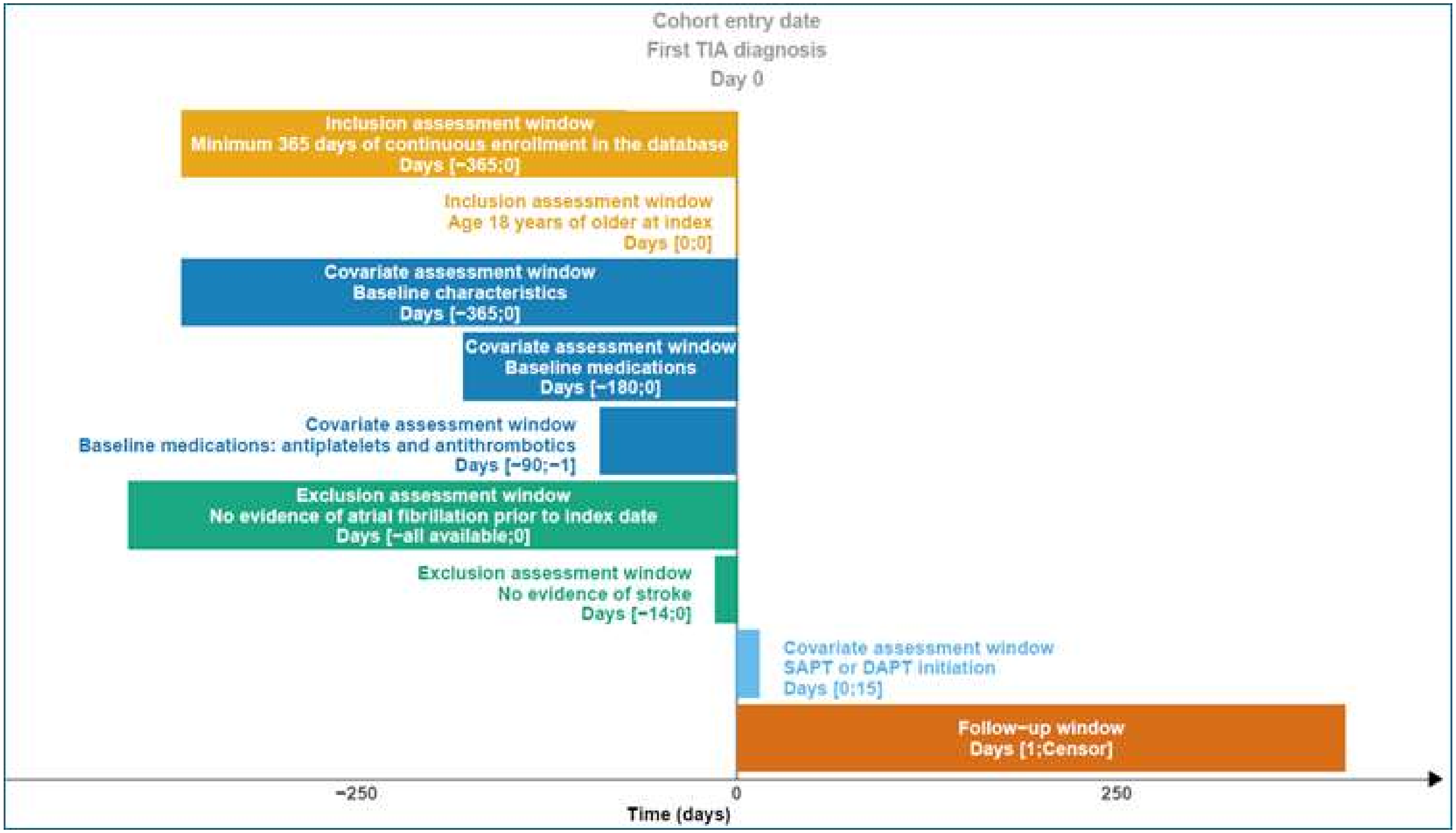

2. Data sources

database	country	cdm_name	cdm_version	cdm_holder	cdm_release_date	vocabulary_version	person_count	observation_period_count	earliest_observation_	latest_observation_	snapshot_date
Merative MarketScan	US	MKTSCAN_CLAIMS_OMOP	5.4	Bayer AG	7/22/2023	v5.0 23-JAN-23	176890290	202823163	1/1/2002	9/30/2022	7/25/2024
Optum Clinformatics	US	OPTUM_CLAIMS_OMOP	5.4	Bayer AG	10/27/2023	v5.0 23-JAN-23	77234542	95612913	1/1/2007	12/31/2022	7/24/2024

3. Medical Codelists

Name	Type	Pre-Studyathon preparation?	Available in Thrombosis Definitions Library?
MC1 - AF	Inclusion/Exclusion	No	Yes
MC2 - TIA	Inclusion/Exclusion	No	Yes
MC3 - Ischemic Stroke	Outcome	Yes	Yes
MC4 - Intracranial bleeding	Outcome	No	Yes
MC5 - Hospitalization-related Bleeding	Outcome	Yes	Yes
MC6 - Hypertension	baseline covariate	No	Yes
MC7 - Myocardial Infarction (MI)	baseline covariate	Yes	Yes
MC8 - Anemia	baseline covariate	No	Yes
MC9 - Stroke	baseline covariate	Yes	Yes
MC10 - Hemorrhagic stroke	baseline covariate	Yes	Yes
MC11 - Hyperlipidemia	baseline covariate	No	Yes
MC12 - Chronic kidney disease (CKD)	baseline covariate	Yes	Yes
MC13 - Heart Failure	baseline covariate	Yes	Yes
MC14 - Chronic Coronary Artery Disease (CAD)	baseline covariate	Yes	Yes
MC15 - Peripheral Artery Disease (PAD)	baseline covariate	Yes	Yes
MC16 - Diabetes mellitus type-1 and type-2	baseline covariate	Yes	Yes
MC17 - Cancer excluding non-melanoma skin cancer	baseline covariate	No	No
MC18 - Dementia	baseline covariate	No	No
MC19 - Venous thromboembolism (VTE)	baseline covariate	Yes	Yes
MC20 - Mechanical heart valves	baseline covariate	Yes	Yes
MC21 - Congenital heart disease	baseline covariate	No	No
MC22 - Patent foramen ovale	baseline covariate	No	No
MC23 - History of Ischemic Stroke	baseline covariate	No	No
MC24 - History of hemorrhagic stroke	baseline covariate	No	No
M1 - Antihypertensives	baseline covariate	No	No
M2 - Antidiabetics	baseline covariate	No	Yes
M3 - Statins	treatment	No	No
M4 - NSAIDs	treatment	Yes	Yes
M5 - Antiplatelet and antithrombotic medications	treatment	No	Yes
M6 - Aspirin	treatment	Yes	Yes
M7 - Clopidogrel	treatment	No	Yes
M8 - Ticagrelor	treatment	No	Yes
M9 - Prasugrel	treatment	No	No
M10 - Cilostazol	treatment	No	No
M11 - Other Antiplatelets	treatment	Yes	Yes
Oral anticoagulants	treatment	Yes	Yes
M 12 - Warfarin	treatment	Yes	Yes
M13 - Direct oral anticoagulants (Rivaroxaban, Dabigatran, Apixaban)	treatment	Yes	Yes
M14 - Rivaroxaban	treatment	Yes	Yes
M15 - Dabigatran	treatment	Yes	Yes
M16 - Apixaban	treatment	Yes	Yes

4. Target and Outcome Cohorts

Name	Criteria	Type
TC1	**Inclusion Criteria**<br>•Adult: 18 years of age or older at index event<br>•First-time TIA: identified as having an inpatient or emergency department record of a diagnostic code compatible with the list of ICD-9 and ICD-10 codes for TIA included in the Annex after 1st January 2012.<br>•Minimum 365 days of continuous enrollment [-365,0]<br>**Exclusion Criteria**<br>•AF: Having evidence of atrial fibrillation (AF) identified as a diagnostic code record compatible with the list of ICD-9 and ICD-10 codes for AF included in the Annex at any time prior to and including the index date.	Base cohort (primary objective)
TC2	base + initiated SAPT between days 0 and 15 after the TIA	Subcohort of base cohort (secondary objective)
TC3	base + initiated DAPT in the 0 to 15 days after the TIA	Subcohort of base cohort (secondary objective)
TC4	base + not initiated SAPT or DAPT in the 0 to 15 days after the TIA	Subcohort of base cohort (secondary objective)
TC5	base + initiated SAPT or DAPT in the 0 to 15 days after the TIA	Subcohort of base cohort (secondary objective)
OC1	base + ischemic stroke in follow up [1; any time[	Outcome cohort
OC2	base + Intracranial bleeding in follow up [1; any time[	Outcome cohort
OC3	base + Hospitalization-related bleeding [1; any time[	Outcome cohort
OC4	base + New-onset atrial fibrillation in follow up [1; any time[	Outcome cohort
OC5	base + Recurrent TIA in follow up [1; any time[	Outcome cohort

5. Medical Concept Set #6417 [REACT-TIA] TIA

Id	Code	Name	Class	Standard Co	Valid Start	Valid End Date	Domain	Vocabulary
4338523	88032003	Amaurosis fugax	Disorder	Standard	1/31/2002	12/31/2099	Condition	SNOMED
36712812	1.2238E+16	Amaurosis fugax of left eye	Disorder	Standard	1/31/2017	12/31/2099	Condition	SNOMED
36712813	1.2238E+16	Amaurosis fugax of right eye	Disorder	Standard	1/31/2017	12/31/2099	Condition	SNOMED
374055	64009001	Basilar artery syndrome	Disorder	Standard	1/31/2002	12/31/2099	Condition	SNOMED
4112020	195200006	Carotid artery syndrome hemispheric	Disorder	Standard	1/31/2002	12/31/2099	Condition	SNOMED
4043734	230716006	Carotid territory transient ischemic attack	Disorder	Standard	1/31/2002	12/31/2099	Condition	SNOMED
381036	73192008	Multiple AND bilateral precerebral artery stenosis	Disorder	Standard	1/31/2002	12/31/2099	Condition	SNOMED
762708	4.3085E+14	Multiple and bilateral precerebral artery stenosis with infarction	Disorder	Standard	9/1/2012	12/31/2099	Condition	SNOMED
765775	4.3086E+14	Multiple and bilateral precerebral artery stenosis without infarction	Disorder	Standard	9/1/2012	12/31/2099	Condition	SNOMED
40485430	444172003	Recurrent transient cerebral ischemic attack	Disorder	Standard	1/31/2010	12/31/2099	Condition	SNOMED
433505	15258001	Subclavian steal syndrome	Disorder	Standard	1/31/2002	12/31/2099	Condition	SNOMED
373503	266257000	Transient cerebral ischemia	Disorder	Standard	1/31/2002	12/31/2099	Condition	SNOMED
4139517	426814001	Transient cerebral ischemia due to atrial fibrillation	Disorder	Standard	7/31/2007	12/31/2099	Condition	SNOMED
37163288	1208871009	Transient ischemic attack co-occurrent with subarachnoid hemorrhage	Disorder	Standard	3/31/2022	12/31/2099	Condition	SNOMED
46272244	710575003	Transient ischemic attack due to embolism	Disorder	Standard	7/31/2015	12/31/2099	Condition	SNOMED
434656	34781003	Vertebral artery syndrome	Disorder	Standard	1/31/2002	12/31/2099	Condition	SNOMED
376714	195199008	Vertebrobasilar artery syndrome	Disorder	Standard	1/31/2002	12/31/2099	Condition	SNOMED
4048785	230717002	Vertebrobasilar territory transient ischemic attack	Disorder	Standard	1/31/2002	12/31/2099	Condition	SNOMED

6. Medical Concept Set #6405 [REACT-TIA] Atrial Fibrillation

Id	Code	Name	Class	Standard C	Valid Start Date	Valid End Date	Domain	Vocabulary
313217	49436004	Atrial fibrillation	Disorder	Standard	1/31/2002	12/31/2099	Condition	SNOMED
37171038	3.13378E+17	Atrial fibrillation due to heart valve disorder	Disorder	Standard	6/30/2023	12/31/2099	Condition	SNOMED
44782442	1.20041E+14	Atrial fibrillation with rapid ventricular response	Disorder	Standard	1/31/2014	12/31/2099	Condition	SNOMED
4141360	426749004	Chronic atrial fibrillation	Disorder	Standard	7/31/2007	12/31/2099	Condition	SNOMED
4117112	300996004	Controlled atrial fibrillation	Disorder	Standard	1/31/2002	12/31/2099	Condition	SNOMED
1340258	OMOP5165913	Exacerbation of atrial fibrillation	Disorder	Standard	5/6/2022	12/31/2099	Condition	OMOP Extension
37395821	715395008	Familial atrial fibrillation	Disorder	Standard	7/31/2016	12/31/2099	Condition	SNOMED
4119601	233910005	Lone atrial fibrillation	Disorder	Standard	1/31/2002	12/31/2099	Condition	SNOMED
45768480	706923002	Longstanding persistent atrial fibrillation	Disorder	Standard	1/31/2015	12/31/2099	Condition	SNOMED
4119602	233911009	Non-rheumatic atrial fibrillation	Disorder	Standard	1/31/2002	12/31/2099	Condition	SNOMED
4154290	282825002	Paroxysmal atrial fibrillation	Disorder	Standard	1/31/2002	12/31/2099	Condition	SNOMED
37172212	4.67644E+17	Paroxysmal atrial fibrillation due to heart valve disorder	Disorder	Standard	6/30/2023	12/31/2099	Condition	SNOMED
605092	1010405004	Paroxysmal atrial fibrillation with rapid ventricular response	Disorder	Standard	1/31/2021	12/31/2099	Condition	SNOMED
4232691	440028005	Permanent atrial fibrillation	Disorder	Standard	1/31/2009	12/31/2099	Condition	SNOMED
37170582	2.80798E+17	Permanent atrial fibrillation due to heart valve disorder	Disorder	Standard	6/30/2023	12/31/2099	Condition	SNOMED
4232697	440059007	Persistent atrial fibrillation	Disorder	Standard	1/31/2009	12/31/2099	Condition	SNOMED
37172244	4.89609E+17	Persistent atrial fibrillation due to heart valve disorder	Disorder	Standard	6/30/2023	12/31/2099	Condition	SNOMED
42539346	762247006	Preexcited atrial fibrillation	Disorder	Standard	1/31/2018	12/31/2099	Condition	SNOMED
4199501	314208002	Rapid atrial fibrillation	Disorder	Standard	1/31/2002	12/31/2099	Condition	SNOMED

7. Medical Concept Set #6473 [REACT-TIA] Ischemic Stroke

Id	Code	Name	Class	Standard Co	Valid Start Date	Valid End Date	Domain	Vocabulary
4138327	426107000	Acute lacunar infarction	Disorder	Standard	7/31/2007	12/31/2099	Condition	SNOMED
4045735	230693009	Anterior cerebral circulation infarction	Disorder	Standard	1/31/2002	12/31/2099	Condition	SNOMED
4031045	14309005	Anterior choroidal artery syndrome	Disorder	Standard	1/31/2002	12/31/2099	Condition	SNOMED
37155661	2.93671E+14	Behavioral disturbance due to multi-infarct dementia	Disorder	Standard	4/30/2022	12/31/2099	Condition	SNOMED
761110	1.51161E+14	Bilateral cerebral infarction due to precererbral arterial occlusion	Disorder	Standard	9/1/2016	12/31/2099	Condition	SNOMED
4110189	195185009	Cerebral infarct due to thrombosis of precerebral arteries	Disorder	Standard	1/31/2002	12/31/2099	Condition	SNOMED
443454	432504007	Cerebral infarction	Disorder	Standard	7/31/2008	12/31/2099	Condition	SNOMED
762951	4.34151E+14	Cerebral infarction due to anterior cerebral artery occlusion	Disorder	Standard	3/1/2013	12/31/2099	Condition	SNOMED
765515	4.34991E+14	Cerebral infarction due to basilar artery stenosis	Disorder	Standard	9/1/2013	12/31/2099	Condition	SNOMED
43530683	1.49821E+14	Cerebral infarction due to carotid artery occlusion	Disorder	Standard	7/31/2013	12/31/2099	Condition	SNOMED
762933	4.33891E+14	Cerebral infarction due to cerebral artery occlusion	Disorder	Standard	3/1/2013	12/31/2099	Condition	SNOMED
762937	4.33961E+14	Cerebral infarction due to cerebral venous thrombosis	Disorder	Standard	3/1/2013	12/31/2099	Condition	SNOMED
4111714	195230003	Cerebral infarction due to cerebral venous thrombosis, non-pyogenic	Disorder	Standard	1/31/2002	12/31/2099	Condition	SNOMED
4108356	195190007	Cerebral infarction due to embolism of cerebral arteries	Disorder	Standard	1/31/2002	12/31/2099	Condition	SNOMED
45772786	705128004	Cerebral infarction due to embolism of middle cerebral artery	Disorder	Standard	1/31/2015	12/31/2099	Condition	SNOMED
4110190	195186005	Cerebral infarction due to embolism of precerebral arteries	Disorder	Standard	1/31/2002	12/31/2099	Condition	SNOMED
762935	4.33931E+14	Cerebral infarction due to internal carotid artery occlusion	Disorder	Standard	3/1/2013	12/31/2099	Condition	SNOMED
763015	4.34961E+14	Cerebral infarction due to middle cerebral artery occlusion	Disorder	Standard	9/1/2013	12/31/2099	Condition	SNOMED
46273649	3.4181E+13	Cerebral infarction due to occlusion of basilar artery	Disorder	Standard	7/31/2015	12/31/2099	Condition	SNOMED
35610084	1.08941E+15	Cerebral infarction due to occlusion of cerebral artery	Disorder	Standard	4/1/2018	12/31/2099	Condition	SNOMED
46270031	1.25081E+14	Cerebral infarction due to occlusion of precerebral artery	Disorder	Standard	7/31/2015	12/31/2099	Condition	SNOMED
762934	4.33911E+14	Cerebral infarction due to posterior cerebral artery occlusion	Disorder	Standard	3/1/2013	12/31/2099	Condition	SNOMED
43531607	9.9451E+13	Cerebral infarction due to stenosis of carotid artery	Disorder	Standard	7/31/2013	12/31/2099	Condition	SNOMED
35610085	1.08942E+15	Cerebral infarction due to stenosis of cerebral artery	Disorder	Standard	4/1/2018	12/31/2099	Condition	SNOMED
46270381	2.93831E+14	Cerebral infarction due to stenosis of precerebral artery	Disorder	Standard	7/31/2015	12/31/2099	Condition	SNOMED
4110192	195189003	Cerebral infarction due to thrombosis of cerebral arteries	Disorder	Standard	1/31/2002	12/31/2099	Condition	SNOMED
45767658	705130002	Cerebral infarction due to thrombosis of middle cerebral artery	Disorder	Standard	1/31/2015	12/31/2099	Condition	SNOMED
44782773	3.4191E+13	Cerebral infarction due to vertebral artery occlusion	Disorder	Standard	1/31/2014	12/31/2099	Condition	SNOMED
46270380	2.93811E+14	Cerebral infarction due to vertebral artery stenosis	Disorder	Standard	7/31/2015	12/31/2099	Condition	SNOMED
37110678	724993002	Cerebral ischemic stroke due to occlusion of extracranial large artery	Disorder	Standard	7/31/2017	12/31/2099	Condition	SNOMED
37110679	724994008	Cerebral ischemic stroke due to stenosis of extracranial large artery	Disorder	Standard	7/31/2017	12/31/2099	Condition	SNOMED
40479572	441526008	Infarct of cerebrum due to iatrogenic cerebrovascular accident	Disorder	Standard	7/31/2009	12/31/2099	Condition	SNOMED
4043731	230692004	Infarction - precerebral	Disorder	Standard	1/31/2002	12/31/2099	Condition	SNOMED
4131383	413102000	Infarction of basal ganglia	Disorder	Standard	7/31/2004	12/31/2099	Condition	SNOMED
4116315	302902003	Infarction of optic chiasm	Disorder	Standard	1/31/2002	12/31/2099	Condition	SNOMED
4046237	230523009	Infarction of optic radiation	Disorder	Standard	1/31/2002	12/31/2099	Condition	SNOMED
4043567	230518009	Infarction of optic tract	Disorder	Standard	1/31/2002	12/31/2099	Condition	SNOMED
4119140	302904002	Infarction of visual cortex	Disorder	Standard	1/31/2002	12/31/2099	Condition	SNOMED
4045740	230702001	Lacunar ataxic hemiparesis	Disorder	Standard	1/31/2002	12/31/2099	Condition	SNOMED

Id	Code	Name	Class	Standard Co	Valid Start Date	Valid End Date	Domain	Vocabulary
42535465	2.92851E+14	Lacunar ataxic hemiparesis of right dominant side	Disorder	Standard	1/31/2018	12/31/2099	Condition	SNOMED
42535524	3.30421E+14	Lacunar ataxic hemiparesis of right nondominant side	Disorder	Standard	1/31/2018	12/31/2099	Condition	SNOMED
4046360	230698000	Lacunar infarction	Disorder	Standard	1/31/2002	12/31/2099	Condition	SNOMED
4141405	307766002	Left sided cerebral infarction	Disorder	Standard	1/31/2002	12/31/2099	Condition	SNOMED
37164462	1231168008	Malignant middle cerebral artery syndrome	Disorder	Standard	6/30/2022	12/31/2099	Condition	SNOMED
4046090	230287006	Mixed cortical and subcortical vascular dementia	Disorder	Standard	1/31/2002	12/31/2099	Condition	SNOMED
379778	56267009	Multi-infarct dementia	Disorder	Standard	1/31/2002	12/31/2099	Condition	SNOMED
37395562	1.06021E+14	Multi-infarct dementia due to atherosclerosis	Disorder	Standard	7/31/2016	12/31/2099	Condition	SNOMED
444091	10349009	Multi-infarct dementia with delirium	Disorder	Standard	1/31/2002	12/31/2099	Condition	SNOMED
443790	25772007	Multi-infarct dementia with delusions	Disorder	Standard	1/31/2002	12/31/2099	Condition	SNOMED
443864	14070001	Multi-infarct dementia with depression	Disorder	Standard	1/31/2002	12/31/2099	Condition	SNOMED
377254	70936005	Multi-infarct dementia, uncomplicated	Disorder	Standard	1/31/2002	12/31/2099	Condition	SNOMED
37116473	733199002	Multifocal cerebral infarction due to and following procedure on cardiovascular s	Disorder	Standard	7/31/2017	12/31/2099	Condition	SNOMED
763009	4.34881E+14	Multiple and bilateral precerebral arterial occlusion with infarction	Disorder	Standard	9/1/2013	12/31/2099	Condition	SNOMED
762708	4.30851E+14	Multiple and bilateral precerebral artery stenosis with infarction	Disorder	Standard	9/1/2012	12/31/2099	Condition	SNOMED
4145897	307363008	Multiple lacunar infarcts	Disorder	Standard	1/31/2002	12/31/2099	Condition	SNOMED
4077086	276219001	Occipital cerebral infarction	Disorder	Standard	1/31/2002	12/31/2099	Condition	SNOMED
765907	4.33941E+14	Occlusion and stenosis of middle cerebral artery with infarction	Disorder	Standard	3/1/2013	12/31/2099	Condition	SNOMED
4046359	230695002	Partial anterior cerebral circulation infarction	Disorder	Standard	1/31/2002	12/31/2099	Condition	SNOMED
4129534	237701005	Pituitary apoplexy	Disorder	Standard	1/31/2002	12/31/2099	Condition	SNOMED
4319146	95830009	Pituitary infarction	Disorder	Standard	1/31/2002	12/31/2099	Condition	SNOMED
4045737	230699008	Pure motor lacunar infarction	Disorder	Standard	1/31/2002	12/31/2099	Condition	SNOMED
4046361	230701008	Pure sensorimotor lacunar infarction	Disorder	Standard	1/31/2002	12/31/2099	Condition	SNOMED
4045738	230700009	Pure sensory lacunar infarction	Disorder	Standard	1/31/2002	12/31/2099	Condition	SNOMED
4146185	307767006	Right sided cerebral infarction	Disorder	Standard	1/31/2002	12/31/2099	Condition	SNOMED
36717605	723082006	Silent cerebral infarct	Disorder	Standard	1/31/2017	12/31/2099	Condition	SNOMED
4142739	427296003	Thalamic infarction	Disorder	Standard	7/31/2007	12/31/2099	Condition	SNOMED
4046358	230694003	Total anterior cerebral circulation infarction	Disorder	Standard	1/31/2002	12/31/2099	Condition	SNOMED
4046089	230285003	Vascular dementia of acute onset	Disorder	Standard	1/31/2002	12/31/2099	Condition	SNOMED

8. REACT-TIA study: Technical and Lay summary

The REACT-TIA study was executed during the studyathon using two US databases mentioned in the manuscript (Optum Clinformatics and MarketScan) but the protocol also contemplates analyses in the UK and Japan, which have been running outside the scope of the studyathon. Therefore, the REACT-TIA study protocol is also registered and approved by CPRD (UK). The technical and lay summaries are publicly available on the CPRD website (**https://www.cprd.com/approved-studies/recurrent-events-and-characteristics-following-first-ever-transient-ischemic)** and provide further background on the clinical research question, study objectives, and methods.
